# Cultural extinction in evolutionary perspective

**DOI:** 10.1017/ehs.2021.25

**Published:** 2021-04-23

**Authors:** Hanzhi Zhang, Ruth Mace

**Affiliations:** Department of Anthropology, University College London, London WC1H 0BW, UK

**Keywords:** Cultural extinction, phylogenetic comparative method, cultural diversity, frequency-dependent selection, human behavioural ecology

## Abstract

Cultural diversity is disappearing quickly. Whilst a phylogenetic approach makes explicit the continuous extinction of cultures, and the generation of new ones, cultural evolutionary changes such as the rise of agriculture or more recently colonisation can cause periods of mass cultural extinction. At the current rate, 90% of languages will become extinct or moribund by the end of this century. Unlike biological extinction, cultural extinction does not necessarily involve genetic extinction or even deaths, but results from the disintegration of a social entity and discontinuation of culture-specific behaviours. Here we propose an analytical framework to examine the phenomenon of cultural extinction. When examined over millennia, extinctions of cultural traits or institutions can be studied in a phylogenetic comparative framework that incorporates archaeological data on ancestral states. Over decades or centuries, cultural extinction can be studied in a behavioural ecology framework to investigate how the fitness consequences of cultural behaviours and population dynamics shift individual behaviours away from the traditional norms. Frequency-dependent costs and benefits are key to understanding both the origin and the loss of cultural diversity. We review recent evolutionary studies that have informed cultural extinction processes and discuss avenues of future studies.

## Graphical abstract

**Figure:**
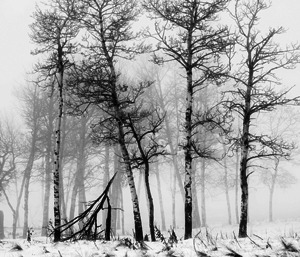

**Social media summary:** Cultural extinction assessed empirically, in phylogenetic histories and in frequency-dependent fitness landscapes.

## 1. Introduction

Languages, like organic beings, can be classed in groups under groups; and they can be classed either naturally according to descent, or artificially by other characters. Dominant languages and dialects spread widely, and lead to the gradual extinction of other tongues. A language, like a species, when once extinct, never (…) reappears. (Charles Darwin, *The Descent of Man*, 1871)

Human societies exhibit extraordinary cultural diversity. There are systematic differences in marital systems, subsistence, political organisation and other social institutions among different cultures, maintained by stable and consistent behavioural and linguistic variations between cultural groups (Barbujani, 1991; Pagel & Mace, 2004). Many aspects of culture leave no trace in history, making it difficult to measure and assess how cultural diversity changed over time. Language, as the primary medium of human cultural learning and transmission, can provide a quantifiable measure of cultural diversity (Loh & Harmon, 2014). Today, about 6,000 languages are spoken around the world (Wurm, 2001). This is much lower than the estimated 12,000 to 20,000 languages spoken worldwide before the spread of agriculture (Pagel, 2009). Among the extant languages, 3,000 or more are classified as endangered (Wurm, 2001). Linguists predict that, at the current rate of language extinction, 90% of languages will become extinct or moribund by the end of this century (Krauss, 1992; Nettle, 1999; Nettle & Romaine, 2000).

**Figure 1.**
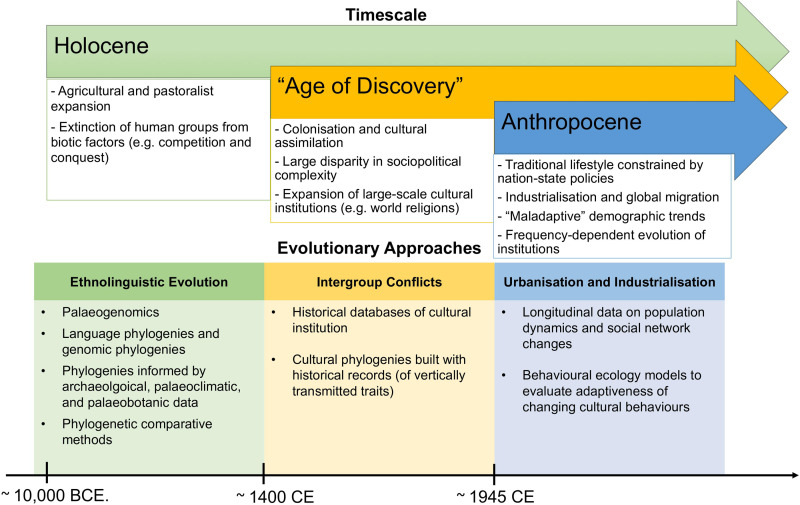
Summary of main evolutionary approaches to study cultural extinction empirically at different timescales, as discussed in this review (we do not claim that these processes only occurred in the time-period mentioned).

The unprecedented scale of cultural extinctions, greatly exceeding the rate of creation of new cultures, is widely appreciated in evolutionary human sciences; yet few studies have examined the phenomena with empirical data. Here we review empirical evolutionary studies to answer two questions: (a) what do we know about cultural extinctions; and (b) how can we study cultural extinction empirically? We will show that cultural extinction since the Holocene can be studied empirically and an evolutionary framework provides a natural framework for thinking about cultural extinction at both macro- and micro-levels (see Figure 1). Anthropology is littered with failed attempts at a widely accepted definition of culture, which makes the definition of cultural extinction equally hard. Without downplaying the complexity of cultural diversity, we propose a working definition for cultural extinction as the loss of cultural phenomena, ranging from individual cultural traits to cultural complexes/institutions, languages and whole ethnolinguistic groups. The definition of cultures includes the shared characteristics and knowledge of a particular group of people; therefore, from an evolutionary perspective, traits like language and kinship systems that show at least some frequency dependence (i.e. when costs and benefits are structured by the frequencies of alternative strategies adopted by others in the population) are likely to be key to both the creation and the loss of cultural diversity. We divide our discussions into two sections – cultural extinction at the macro-evolutionary level and the micro-evolutionary level. At the end of each section, we discuss methodological challenges and directions of future studies.

## 2. Macro-evolutionary view of cultural extinction

Like biological species, cultural groups are subject to hereditary transmission and variation by mutation and selection – the prerequisites of evolutionary changes. Ethnolinguistic diversity evolves in ways similar to biological speciation (Collard, Shennan, & Tehrani, 2006). Descendant groups split from the ancestral group and, over time, evolve new customs and rules independently while preserving some of the inherited practices (Pagel & Mace, 2004). Communication between individuals of different groups is often impeded by ecological boundaries, language barriers, endogamy and xenophobic prejudices (Barth, 1969; McElreath, Boyd, & Richerson, 2003). Notably, language, marriage patterns and xenophobia are all likely to be frequency-dependent traits, which may therefore favour human social organisation to cluster into cultural groups. It is therefore possible to reconstruct the evolutionary history of human populations and cultural groups using archaeological, genomic (especially ancient DNA) and most often language data (Mace & Pagel, 1994).

Below we review how genomic and linguistic phylogenies helped identify cultural extinction, and how a phylogenetic approach can answer questions relating to historical cultural extinction processes, the role of ecological and intrinsic factors in cultural extinction, and whether cultural extinction occurs by replacement, admixture or cultural diffusion. We then describe a case study that combined historical records with phylogenetic comparative methods to empirically examine the extinction of religious groups. Finally, we discuss methodological limitations of studying cultural extinction empirically and promising avenues for future research.

### Identifying cultural extinctions in prehistory

Here we discuss how cases of ethnolinguistic extinction in prehistory can be identified with limited empirical evidence. A frequently observed pattern of biodiversity is phylogenetic imbalances caused by disparities of clade sizes (i.e. the number of extant taxa in a clade; Heard & Mooers, 2002; Ricklefs, 2007). Although variation in clade size is expected owing to purely stochastic processes of diversification turnover, many families exhibit an imbalance far beyond what is expected by chance alone (Mooers & Heard, 1997) and reflect true differences in speciation and/or extinction rates. Imbalances in cultural phylogenies of ethnolinguistic groups can provide clues about past extinctions. In extreme cases, we observe linguistic and genetic ‘isolates’ with no close relatives among extant ethnolinguistic groups. Assuming that linguistic diversity turnovers are similar among clades (which is a big assumption), an isolate group or clade suggests severe disruption to ethnolinguistic turnover in recent history and probable mass extinctions in deep history.

The Basque language (Euskara), for instance, is one of the few remaining languages left in Europe that is not Indo-European in origin and does not seem to be linked with any other major language families (Kurlansky, 1999). Genetically, the Basque people also show some differences from other European populations (Behar et al., 2012). Some believe that Basque represents a relic of ancient, pre-agricultural linguistic diversity in Europe, with roots as far back as the Paleolithic hunter–gatherer populations, although recent discoveries (Gunther et al., 2015) of close links between modern Basque and early Iberian farmers suggest that the evolutionary history of the Basque culture is likely to be more complex.

The Nivkhi language represents another relic linguistic lineage outside the world's major language families, with no demonstrable genealogical relation to either neighbouring or geographically distant languages (Georg, 2018). Most of their traditional territory was dramatically reshaped by the expansion of Tungusic-speaking groups. Genetic phylogenies of relic groups of the Russian Far East (Dryomov, Starikovskaya, Nazhmidenova, Morozov, & Sukernik, 2020) – including Nivkhi, Oroki, Koryak and Udegey – found genetic continuity of these groups with late Pleistocene hunter–gatherers, albeit varying degrees of genetic admixtures with external groups. The existence of genetic/linguistic isolates clearly demonstrates that the process of cultural extinction has occurred throughout human history.

### Neolithic farming expansions and ecological constraints

What could have triggered mass extinctions of prehistoric cultures? Many believe that the geographically uneven development of food production gave the first agricultural groups advantages over hunter–gatherer societies and caused the dispersal of agriculturalists along with their languages and lifestyles into new territories (Heggarty et al., 2010), hastening the disappearance of hunter–gatherer cultures. Phylogenetic inferences support this hypothesis in some language families (R. Bouckaert et al., 2012; Grollemund et al., 2015; Zhang, Ji, Pagel, & Mace, 2020), although not in others (R. R. Bouckaert, Bowern, & Atkinson, 2018; Chang, Hall, Cathcart, & Garrett, 2015). Ecology also played a key role in facilitating the first dispersals of some populations. The expansions of agricultural groups may be triggered by the deteriorating homeland environment for early farmers, as seen in the cases of Greece (Van Andel, Zangger, & Demitrack, 1990) and the Levant (Rollefson & Köhler-Rollefson, 1993), both of which suffered severe environmental damage during the Neolithic. Physical ecology also seems to constrain the migration and dispersal routes of ethnolinguistic groups. Phylogenetic reconstruction of the Bantu language family, informed by geographical data and palaeoclimatic records of sub-Saharan regions, shows that, when ancestral Bantu populations expanded from savannah homeland in West Africa, they avoided unfamiliar rainforest habitat and took advantage of a savannah corridor through the Congo rainforest that emerged briefly owing to climate change in the western Congo basin (Grollemund et al., 2015). The authors showed that dispersal rates slow down when ancestral populations transition from savannah habitat into rainforest habitat. Geographical phylogenetic reconstruction of the Pama–Nyungan language family also showed that dispersal rates were two times slower near water, supporting a link between language spread and ecological factors associated with mobility and range size (R. R. Bouckaert et al., 2018).

### Prehistoric cultural extinctions by replacement and admixture

Occasionally, archaeological remains provide direct evidence for historical human populations and clues as to how they went extinct (e.g. by replacement or assimilation). Ancient genomics can contribute knowledge about now-extinct cultures, from which little is known about their phenotypic traits, genetic origin and biological relationship to present-day populations. For instance, a Paleo-Eskimo human fossil in Greenland from the early Saqqaq settlement (3,900–2,500 BP; Gilbert et al., 2008) revealed that the earliest migrants to the New Word's northern extremes derived from populations in the Bering Sea area and were not directly related to Native Americans or the later Neo-Eskimos that replaced them. In South America, ancient-DNA (Posth et al., 2018b) and morphological (Hubbe, Okumura, Bernardo, & Neves, 2014) analyses point to a population turnover more than 9,000 years ago, when populations of the Clovis culture replaced the indigenous people at that time. In Africa, genomic records suggest that the prehistoric African population structure was largely reshaped by the expansion of Bantu farmers from Western Africa who displaced indigenous forager populations in Eastern Africa, and later by the movements of pastoralists from eastern to southern Africa (Skoglund et al., 2017). The lineage of individuals living in eastern African 4,500 years before present (BP) is preserved in contemporary Hadza populations in Tanzania but appears to have contributed little ancestry to present-day Bantu speakers in eastern Africa, who instead trace their ancestry to a lineage related to present-day western Africans. Population replacement by incoming farmers and pastoralists appears to have been nearly complete in Malawi, where ancestry from the indigenous populations from 8,100–2,500 years BP were almost completely replaced by Bantu population of western African origin as detected in present-day Malawian (Skoglund et al., 2017).

In Europe, westward migration of so-called Yamnaya Steppe herders from eastern Europe in the Early Bronze Age (around 2,500 BCE) was so massive that it led to the large-scale replacement of populations in central Europe – the Corded Ware people in Germany around that time show as much as 75% Yamnaya ancestry. The Yamnaya also spread eastward from the Steppe and into the Altai region of southern Siberia, founding the Afanasevo Culture, which shows an almost uniquely Yamnaya ancestral profile, suggesting a large-scale population replacement rather than admixture with the local population (Allentoft et al., 2015). A similar scenario probably occurred in northern China during the second millennium BC. It seems plausible that the Neolithic farmers of northern China faced similar profound immigration pressure at about the same time as their counterparts in central Europe. A recent study of demographic modelling (Leipe, Long, Sergusheva, Wagner, & Tarasov, 2019) suggests that, after ca. 2,000 BCE, the observed decline of farming populations in different parts of north-central China was probably the result of enhanced expansions of agropastoralists leading to competition with indigenous farmers for natural resources (e.g. copper and pastoral grounds) and the spread of plague epidemics (Hosner, Wagner, Tarasov, Chen, & Leipe, 2016).

Interestingly, the genetic influx of the Yamnaya in central Europe does not appear to be protracted over a very long time and started to decline after the initial Bronze Age, as the genetic lineages of the indigenous hunter–gatherers gradually increased (Haak et al., 2015). This suggests that the migration from the Steppe was a short-term event rather than protracted gene flows. The subsequent genetic resurgence of local populations also suggests that European foragers were not completely replaced by the incoming Steppe herders but had extensive admixture with them. In Africa, genomic analyses show that local Berber populations were already admixed with Europeans before the Roman conquest (Fregel et al., 2018). In South-East Asia after ca. 4,000 years BP, local populations admixed with multiple incoming waves of East Asian migration of Austroasiatic, Kradai and Austronesian speakers without being replaced by migrant populations (McColl et al., 2018). Recent genomic and archaeological findings revealed intricacies in cultural extinctions by population replacement and call for a rethinking of frameworks modelling universal cultural-change processes at the population level.

### Prehistoric cultural extinction by mass immigration and social contact

Unlike extinctions in biological evolution, extinctions of local cultural practices following the adoption of foreign cultural practices can take place without population replacement or movement between groups. Contrasting the genetic records with archaeological records of populations in Ice Age Europe shows that similar material cultures of Venus figurines are associated with two populations of the Věstonice Cluster and the Mal'ta, albeit there is no genetic connection between the two, probably reflecting diffusions of ideas without the movements of people (Fu et al., 2016). Cultural diffusion may have been the dominant process of neolithisation in regions of the Baltic and northeastern Europe, where indigenous hunter–gatherers adopted the Neolithic culture gradually. Scandinavian Neolithic hunter–gatherer fossils showed little or no evidence of admixture with neighbouring farmers, despite having coexisted for up to 40 generations in the same region (Skoglund et al., 2014). Similarly, unlike Neolithic farmers in central Europe who show a notable Anatolian component (Omrak et al., 2016), no Anatolian component was detected in the Neolithic genomes from either Latvia or Ukraine (Jones et al., 2017), indicating that the transition to a Neolithic way of life was not produced by considerable immigration of foreign groups. In the Himalayan region, marked changes in material culture and mortuary rituals associated with the Mebrak and Samdzong periods were observed despite long-term continuity of the genetic lineage of local populations (which suggests little population admixture) from 3,150 to 1,250 years BP (Jeong et al., 2016).

Recent advances in archaeological and genetic studies of human prehistory revealed that, in most cases, indigenous people played an active role in the transition to a farming lifestyle and both cultural and demic diffusions were involved. Certain regions have stronger evidence of indigenous adoption than others do (Price, 2000). For instance, genomic records suggest that the Neolithisation of the North Africa population involved the movement of both ideas and people, with Maghrebi populations showing long-term genetic continuity from the early Neolithic to contemporary times and Late Neolithic Moroccans showing genetic admixture with Iberian farmers (Fregel et al., 2018). The spread of farmers and in sub-Saharan Africa from West Africa and savanna pastoralist from East Africa results in a variety of outcomes ranging from no detectable admixture in present-day populations to substantial admixture with previously established hunter–gatherers (Skoglund et al., 2017). Thus, empirical investigations of the balance between demic and cultural diffusion (i.e. indigenous adoption) in cultural extinctions during the Neolithic transition should be approached on a case-by-case and region-by-region basis. Recent models combining demic and cultural diffusion have been developed (Fort, 2015) with some emphasising the interactions between farmers and indigenous hunter–gatherers, particularly at frontier zone, leading to indigenous adoption of a Neolithic way of life.

### Are there intrinsic determinants of cultural extinction?

Cultures, like species, are always dying out. In biological evolution, interspecific competition can influence the dynamics of geographic range size evolution, which influences the formation of incipient species (Phillimore et al., 2007; Rundell & Price, 2009) and their persistence in time (Harnik, Simpson, & Payne, 2012; Jablonski, 2008). Cultural groups also compete for access to land and resources. Warfare was common in human societies long before we adopted agriculture and sedentary lifestyles (Keeley, 1996). In New Guinea, empirically estimated rates of clan extinction among horticulturalists were 10% every 25 years (Soltis, Boyd, & Richerson, 1995). Ethnographic, historical and archaeological records (Bowles, 2009; Keeley, 1996; Soltis et al., 1995) showed that, when a cultural group is defeated in warfare, the social unit may cease to exist, but its surviving members may quickly integrate themselves into the winning group. Cultural traits of the successful groups often spread and the cultural traits of defeated groups and polities often decline. Similarly, groups with institutions that more effectively foster cooperation are most likely to be the victor (Turchin, 2016). Victorious groups often expand their borders and absorb conquered peoples.

Cultural assimilation may be passive, in which defeated people adopt the identity of the dominant group through marriage and/or migration, or it can be coercive. For instance, slaves captured by the Comanche, and their subsequent offspring, became Comanche themselves after one or two generations (Hämäläinen, 2001). Women or other prisoners are often taken as trophies of war. If they marry into the victorious culture, the cultural inheritance of the winning group is mostly unaffected even when their genes are mixing with other groups (Renfrew, 1990). Militarily successful cultures such as the Mongols (Turchin, 2006) and Nuer (Kelly, 1985) often assimilated defeated groups. Cultural systems associated with Christianity and Islam have also spread partly through military conquests, facilitated by both coercive and voluntary conversion of peoples in the defeated group (Richerson et al., 2016).

In many cases, the outcomes of intergroup competitions were determined by intrinsic factors such as sociopolitical complexity and technological innovations. Following the advent of food-producing subsistence, the increasing disparities in sociopolitical complexity underline the human history of conquest and colonisation. Variations in social organisation confer a greater military advantage to centralised groups than to isolated exogamous clans, allowing more centralised communities to conquer and expand their territories. A phylogenetic comparative study of political complexity among Austronesian-speaking societies (Currie, Greenhill, Gray, Hasegawa, & Mace, 2010) showed that, in the most likely scenario, the proto-Austronesian society was acephalous and became more hierarchical as the population moved through the islands of South-East Asia, but then some groups became less hierarchical after entering the region around New Guinea, probably owing to founder effects, contact with the indigenous population or ecological factors.

Based on archaeological evidence, migrations of the Yamnaya from the Steppe into Europe is believed to have been aided by the development of horse riding and the invention of chariots, which also brought the Kurgan burial mounds to Europe (Haak et al., 2015). While most language phylogenies attribute the initial spread of major language families to the advent of agriculture or horse pastoralism, a recent phylogenetic reconstruction of Pama–Nyungan languages (R. R. Bouckaert et al., 2018) presented a unique case of hunter–gatherer language expansion, which the authors argue was facilitated by technological breakthroughs (e.g. new tools and extractive technologies) and social innovations (e.g. patrilineal kinship, exogamous marriage and multigroup rituals) that enabled assimilation (rather than replacement) of the existing hunter–gatherer groups in the marginal environments of Australia 4,000–5,000 years BP. These findings echo another phylogenetic study (Gray, Drummond, & Greenhill, 2009), which reveals a series of settlement pauses and expansion pulses linked to technological and social innovations as ancestral Austronesian speakers expanded from their homeland in Taiwan to the Pacific islands.

Social institutions that promote cooperation (e.g. religious systems) may also contribute to varying competitiveness among cultural groups (see Smith, 2020 for a review of whether cultural group selection might be occurring in such cases). For instance, empirical analyses of the longevities of nineteenth-century communes (Sosis & Bressler, 2003) found that, overall, religious communes with more costly constraints are likely to survive longer than secular communes with less costly constraints. Rigid, inflexible rules and ideologies that cannot adapt to changes in the selective environment may contribute to greater risks of cultural extinction. The demise of Latin, for example, exemplifies how standardisation can limit the available options of prestigious languages and reduced intelligibility (Wright, 2004). In contrast, the unstandardised *lingua franca* may be more flexible in response to the different environment and functional demands.

### Group extinctions on the phylogeny of religious history

**Figure 2.**
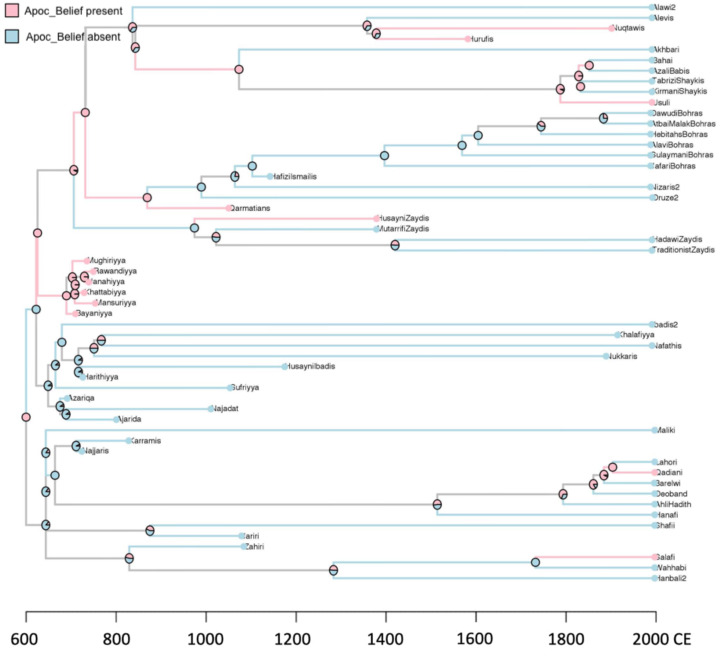
Reconstructed ancestral states of apocalyptic belief for group survival analyses, from Basava et al. (2021, Supplementary Information). Coloured branches indicate the character state a branch ends in, when the tip/node the branch leads to a known state. Grey branches lead to internal nodes with uncertain character states. To assess the potential impact of beliefs and violence on the longevity of Islamic sects, the authors assessed whether the ending state of a branch predicts its length using time-to-event analyses. Branches leading to an extinct group were recorded as an event. Branches leading to a bifurcating node or a contemporary group at the tips were recorded as right-censored, as extinction events may take place at times beyond their endings.

The extinction of cultural traits and groups is often hard to study directly if the evidence is not ‘fossilised’ in the archaeological record, and phylogenetic inference from extant populations can leave extinct groups hidden. However, cultural extinction can be studied more directly in the historical period. The fairly complete historical record of world religion provided a unique opportunity to reconstruct the evolutionary history of religious subgroups on a phylogeny calibrated by timings of splitting and extinction events that were recorded in written history. In a recent study, Basava, Zhang, & Mace (2021) reconstructed the cultural phylogeny of historic Islamic sects in the seventh to twentieth centuries based on written historical records of sect ancestry, presence of beliefs in extant sects as well as historical sects, incorporated into the phylogeny as ‘fossils’ on internal nodes to informs comparative inferences (see Figure 2). They assessed the relationship between afterlife beliefs and the longevity of sects using time-to-event analyses of branch lengths (i.e. empirically recorded duration of sect survival). They found that, among historical Islamic sects, beliefs in an imminent apocalypse predict accelerated sect extinction, even after phylogenetic associations are controlled. Nevertheless, a causal relationship between group extinctions and the apocalyptic belief (or any other intrinsic characteristic of cultural groups) could not be determined without controlling for a wide range of socioecological factors.

## Discussion

Phylogenetic studies revealed that cultural extinctions were not necessarily driven by large, complex groups replacing populations of smaller cultures via warfare and conquests; they can happen as the result of admixtures and social contact between populations. Interdisciplinary research combining current and ancient genomics, archaeology, anthropology and other approaches can provide a more detailed picture of cultural and population turnover (Sjögren et al., 2020; Gokcumen & Frachetti, 2020). For instance, the Hadza and the Sandawe hunter–gatherers are speakers of Khoisan languages, but their genomes are distinct from South African Khoisan populations and show signs of admixtures with neighbouring Bantu farmers. In this case, although Hadza and the Sandawe were socially separated from Bantu and preserved their native languages, the social barriers did not seem to prevent genetic exchanges with their farming neighbours (Cavalli-Sforza, 2000). On rare occasions, language continuity can be preserved after population replacement. For instance, ancient DNAs reveal that subsequently arrived Papuan languages did not replace Austronesian languages in Remote Oceania despite the massive demographic change (Posth et al., 2018a). In some cases, the genetic replacement is facilitated by sex-biased interactions; for example, genomic analyses of Gaucho in southern Brazil found strong Spanish influence of the patriline while matriline materials mainly came from the extinct Charrua culture as well as extant Guarani tribes (Marrero et al., 2007). Alternatively, language replacement can occur with little gene replacement. Celtic languages were replaced relatively rapidly by English in Scotland during the nineteenth and twentieth centuries (Kandler, Unger, & Steele, 2010). The Finns speak a Uralic language but they have very few Uralic genes. Some believe that a very small group of farmers first settled in Finland, perhaps 2,000 years ago; later waves of settlers had peaceful contact with native inhabitants and adopted the natives’ language, which facilitated their settlement and dispersal in the region, albeit little genetic exchange between the two (Cavalli-Sforza, 2000). In short, biological history does not always mirror cultural history.

Ancient genomic material from skeletal remains and both autosomal and uniparental markers will continue to allow testing of the extent to which cultures were spread by people (demic diffusion), or a result of local adoption without incoming migrants (cultural diffusion), as well as the role that admixture plays in culture change and the extent to which independent invention and cultural convergence have occurred. However, caution is needed in equating change in ancient genome profiles of past populations with changes in material culture over time. We cannot assume that individuals or populations sharing certain cultures belonged to closely related populations, as shown by the recent genomic analysis of the Bell–Beaker complex of Europe (4,750–3,800 years) that spread from Iberia into central Europe by cultural diffusion, but later dispersals show strong evidence of demic diffusion, replacing approximately 90% of Britain's gene pool within a few hundred years (Olalde et al., 2018).

Cultural comparative studies are increasingly taking account of the pitfalls of Galton's problem (Mace & Pagel, 1994) and seek to account for the phylogenetic association. Linguistic, ethnographic, historical and ecological datasets such as D-place (Kirby et al., 2016), eHRAF (Murdock, 1983), Seshat (Turchin et al., 2015) and the Database of Religious History (Slingerland & Sullivan, 2017) allow researchers to conduct phylogenetic comparative studies on published phylogenies of major language families in the world, including Austronesian (Gray et al., 2009), Indo-European (R. Bouckaert et al., 2012), Pama–Nyungan (R. R. Bouckaert et al., 2018), Dravidian (Kolipakam et al., 2018), Sino-Tibetan (Zhang et al., 2020), Semitic (Kitchen, Ehret, Assefa, & Mulligan, 2009), Japonic (Lee & Hasegawa, 2011), Turkic (Hruschka et al., 2015), Uralic (Honkola et al., 2013), Uto-Aztec (Dunn, Terrill, Reesink, Foley, & Levinson, 2005) and Bantu languages (Grollemund et al., 2015). The development of comparative cultural databases also calls for new methodologies tailored to these datasets – often limited in sample size and inherently uncertain – to answer the big questions about cultural extinction. Phylogenetic studies are strengthened when they incorporate empirical data from the archaeological and historical records to inform their inferences. Some methods of controlling phylogenetic associations can obscure the uncertainties in phylogenetic reconstructions, for instance, by reducing the posterior sample of inferred language phylogenies to a single matrix of ‘average’ phylogenetic distance between pairs of cultures in a regression model. Other approaches that discount the lineage-specificity of cultural evolutionary processes (e.g. supertree fusing different language families near the root) should also be applied with caution, especially with traits that have been empirically demonstrated to follow lineage-specific evolutionary trajectories (e.g. Dunn, Greenhill, Levinson, & Gray, 2011; Passmore & Jordan, 2020).

Theoretical studies in evolutionary biology offer an abundance of hypotheses (e.g. trait-dependent extinction, evolutionary dead-ends) regarding the global process of extinction. However, research on cultural extinction at the macro-evolutionary level is at a relatively early stage, as it seeks to incorporate insights and empirical evidence from genetics, linguistics, archaeology, anthropology, history and other social sciences, to formally test hypotheses about the drivers and patterns of cultural extinction process.

## 3. Micro-evolutionary view of cultural extinction

In this section we review empirical findings on individuals in endangered cultures, including descriptive studies of the impact on them of social and ecological changes associated with the endangerment of their cultures, and how evolutionary studies can examine behavioural shifts against the background of a changing fitness landscape.

### Social and ecological aspects of the endangering of cultures

The historical and socio-political background of recent cultural extinction events is complex. It is important for evolutionary studies to acknowledge institutional influences on endangered cultures and the reality of individuals living in an endangered culture when modelling the fitness landscape during the cultural extinction process. European colonisation since the sixteenth century has greatly accelerated the rate of extinction of indigenous cultures (Bodley, 1990; Burger, 1987), both directly through warfare and indirectly through social and ecological changes. In most cases, the arrival of foreign settlers reshaped the entire ecosystem by introducing non-native livestock, crops, and bacteria and viruses to the colonies. Historical records showed that, in the nineteenth century, many marginal subsistence producers did not benefit from the market but were forced by the market into the progressive deterioration of production conditions after losing their property rights; incipient market integration in the late Victorian era may have contributed to growing social vulnerability to climate changes and large-scale subsistence crises in many parts of the world (e.g. mass famine and disease epidemics in south Asia, north China, northeast Brazil, and southern Africa; Davis, 2002). Forced market integration could lead to breakdowns of traditional subsistence and social networks and worsen inequality of access to technology and economic participation. For instance, historical records showed that the British rule emancipated local political chiefs from the obligation to invest in community resources; in Gujarat, the new property forms freed village caste-elites from traditional reciprocities and encouraged them to exploit irrigation resources to their selfish advantage (Hardiman, 1998).

Most indigenous people still living in traditional ways on their ancestral lands speak endangered languages, if their native language is not already extinct. Along with the languages, the traditional knowledge of means of livelihood, land use and natural resource management, and various cultural beliefs associated with subsistence, are also being lost (Loh & Harmon, 2014; Salali et al., 2020). Most small-scale indigenous societies are losing the means or numbers to survive, as societies that have more political complexity (i.e. nation-states) dominate the production and consumption interdependencies in the global economy. Among those groups that managed to adapt to the expansion of centralised government and the market, significant sociopolitical changes were accompanied by the extinction of cultural practices associated with traditionally adaptive strategies (Johnson & Earle, 2000). The development of nation-states – accompanied by the prevalence of literacy, education and communication, and the standardisation of speech – also places minority cultural traits under pressure and in many cases has led to their decline and cultural extinction (Heggarty, 2007).

Some argue that the free market promotes values solely based on supply-and-demand, individualism, class inequalities, exclusive policies and excessive consumption while eroding the social bonds in traditional societies (Johnson & Earle, 2000). Empirical observations suggest that the impacts of market integration on indigenous populations are heterogeneous. For instance, in Brazil, the informal British colonialism in the nineteenth century did not affect all regions equally; while the northeastern sugar *fazendas* grew dependent upon British capital, the southern coffee industry was more independent (Deutsch, 1996). Market integration of indigenous populations in the twentieth century seems to have radically transformed traditional cooperative networks in some groups (e.g. Machiguenga forager–horticulturalists (Henrich, 1997) and Kalahari !Kung (Yellen, 1990)), yet in other groups (e.g. Tsimane Amerindians (Gurven et al., 2015) and Huaraoni forager–horticulturalists (Franzen and Eaves, 2007)), there is little evidence that market integration disrupted or reshaped traditional cooperative networks.

Historically, proximity and contact with external groups do not inevitably lead to the demise of foraging societies. ‘Hunter–gatherers’ living in endangered cultures today are often portrayed in media as relics of isolated populations who had no interaction with food-producing populations. This is a misconception given the abundance of historical records and ethnographies showing that many foraging populations have been living in proximity and symbiosis with food-producing populations for thousands of years, have complex, long-range trade networks and have participated in minor food production since prehistoric times, long before the arrival of Europeans in the sixteenth century (Headland & Headland, 1997). So what is different about their contact with external cultures in the last century that led to the unprecedented rates of indigenous language and culture extinction?

### Changing fitness landscape in endangered cultures

Behavioural ecology models make explicit that the optimal behavioural responses of individuals depend on the ecological context. In many cases, preserving the traditional way of life in a non-traditional environment can become costly to survival and reproductive success. Human behavioural ecology studies have shown that cultural behaviours like marriage systems (Thomas et al., 2018), religious rituals (Ge, Chen, Wu, & Mace, 2019; Power, 2017) and taboos (Colding & Folke, 2001) are intricately linked with the local cooperative network that evolved as an adaptation to the local ecology. With socioecological changes, the fitness landscape can also change and render traditional cultural practices/institutions maladaptive in the new environment. Combining empirical data with testable evolutionary hypotheses, human behavioural ecology provides a useful framework to understand individual behaviours within an endangered cultural group, including seemingly maladaptive behaviours, in response to dramatic socioecological changes precipitating cultural extinction. Optimal decisions maximise fitness given relevant trade-offs. Behavioural ecology models can theoretically unite different currencies, such as mortality risk and economic benefit, through their impact on the common currency of reproductive success, or some proxy such as long-term survival.

A behavioural ecology framework helps identify maladaptive behaviours when the socioecology is in flux, as observed among the Casiguran Agta in the Philippines. From the 1960s to 1990s, the Casiguran Agta hunter–gatherers in the Philippines experienced population decline owing to high mortality rates following an influx of many thousands of immigrants into their area, deforestation, depletion of traditional game and plant resources, rising alcoholism, new forces introducing general poverty and new diseases, and cases of outright land-grabbing, murders and kidnappings. With their important traditional resources depleted, the Casiguran Agta have modified their economic behaviour: hunting has declined and wage labour has increased. Trends of population decline were exacerbated by the marrying out of reproductive women; one-third of Agta reproductive females married non-Agta partners and another third out-migrated from traditional areas for wage-labour, leaving behind a high number of unmarried Agta men without potential marriage partners. This pattern of female hypergyny in Casiguran Agta increased significantly in the second half of the last century, which contributed to the eventual extinction of most populations of this small ethnic group. Ethnographers (Headland & Headland, 1997) noted ‘We may logically conclude that these changes have moved the Agta population to a maladaptive state today, manifested by increased high death rates and population decline. Their tragic morbidity and mortality figures could not have been as high in the past as they are now, or the population would have gone extinct long ago.’ The specific communities studied by the Headlands no longer exist. Similar patterns are also found in other populations, such as the Mukogodo; there is ethnographic evidence (Cronk, 2002) that Mukogodo foragers experienced a shortage of wives as they could not compete with wealthier pastoralist Samburu men as Mukogodo women married out of the group.

Social/kinship networks probably mediate the impact of sociopolitical changes on cultural extinction. Traditional societies rely heavily on kin networks to coordinate social and ecological action (Apicella, Marlowe, Fowler, & Christakis, 2012; Berté, 1988), as cooperation with kin is less prone to free-riding (Hughes, 1988). Human evolved as ‘cooperative breeders’, relying on relatives (Hrdy, 2011) and unrelated individuals in the group (Page et al., 2019) for support of reproduction. In many traditional societies, labour exchange is often given freely between kin, but exchanges between unrelated members of a community often require explicit or implicit willingness to reciprocate (Berté, 1988). For many traditional societies transitioning into the market economy, there is an evident tension between the desire to maintain a traditional lifestyle and the perceived benefits of the market economy (Colleran, 2016; Salali et al., 2020). Empirical data showed that individuals in these societies reported declining kin prominence in their social networks and even antagonistic kin relations as their needs become better fulfilled by contacts outside the family (Kasper & Borgerhoff Mulder, 2015). Dense social networks generate social interdependence and rapid consensus formation but also social control and resistance to change. Increasing non-kin interactions could disrupt these evolved patterns of coordination (Newson, Postmes, Lea, & Webley, 2005). Non-kin interactions may allow the spread of new values, as horizontal transmission scales up over vertical transmission (Cavalli-Sforza & Feldman, 1981). Demographic models showed that, for subsistence cultures that depend on effective kin cooperation, falling fertility creates a crisis when it results in too few kin to join the community project (David-Barrett & Dunbar, 2017). Societies may transition to small effective kin networks owing to falling fertility, increased physical distance to kin (e.g. urbanisation) or high mortality (e.g. war or epidemics). These small kin networks will only be able to remain socially cohesive if they replace disappearing kin networks with non-related alternatives (David-Barrett & Dunbar, 2017). Otherwise, sparser networks with diverse, weak, cross-cutting connections can spread novel information easily and quickly within a community, help reject existing social hierarchies and accumulate cultural innovations across communities through partial connectivity (Derex & Boyd, 2016). Empirical data on social networks during cultural extinction would further elucidate this process.

The breakdown of traditional subsistence and social networks can lead to pervasive feelings of dislocation and mental health crises in native communities. Many indigenous cultures in nation-states were plagued by epidemics of substance abuse and mental health crises which have led to staggering rates of unnatural deaths in recent decades (see review in Ohenjo et al., 2006). Some anthropologists believe that substance abuse was responsible for the deteriorating mental health and increasing violence and deaths in post-contact indigenous cultures including the Māori in New Zealand (Baxter et al., 2006), the Hadza of Tanzania (Marlowe, 2010), the San in Botswana (Ikeya, 2002), aboriginal Australians (Pearson, 2001), Inuit societies in Canada (Seale, Shellenberger, & Spence, 2006), the Sami reindeer-herders in Sweden (Ahlm, Hassler, Sjölander, & Eriksson, 2010), and many other indigenous cultures. Mental health issues are also a fundamental issue as we also observe high mortality not directly related to substance abuse. In the Guaraní Kaiowá in Brazil, 69 cases of suicide were recorded from the population of 24,000, 25 times the national average suicide rates of the Brazilian population; many were young people who died by drinking poison or hanging themselves (Coloma, Hoffman, & Crosby, 2006). If the post-contact foraging populations suffered from an epidemic of substance abuse and mental health crises, it is unclear whether their excess adult mortality is an unprecedented phenomenon that only originated in recent decades.

### Extinction of cultural norms by frequency-dependent selection

Cultural extinction is now accelerating all over the world, as cultural groups become more connected to groups with technologies that may out-compete traditional adaptations. In some cases, cultural extinction is the consequence of aggregated behavioural changes structured by demography, often in the face of overwhelming asymmetries in political power or technology. Cultural homogenisation occurs following extensive horizontal transmission between two cultural groups. Cultural shifts, often accompanied by large-scale demographic replacement, are often biased towards the politically dominant culture. This process is often influenced by frequency-dependent transmission biases (Boyd & Richerson, 1985) and/or frequency-dependent costs and benefits, as well as local demographic dynamics. Models and empirical findings (Borenstein, Kendal, & Feldman, 2006; Ji et al., 2016) have shown that the changing frequency of cultural traits can disrupt local evolutionary equilibriums and reshape social interaction networks leading to a cascade of cultural traits being transmitted as individuals tend to conform to the local norm. The relative size of populations, political complexity (Currie & Mace, 2009) and connectivity of kinship networks may determine the extent and direction of cultural diffusion at borders where individuals of different cultural groups live close to each other.

Cultural traits are rarely neutral and payoffs (and hence selection pressures) in social traits are often frequency dependent. Frequency-dependent selection plays a role in cultural homogenisation and extinction in those social traits where coordination with other group members influences costs and benefits. Kandler et al (2010) provide an empirical example using languages, as a particular language is clearly a trait that is only of benefit if those around you also speak the same language. A theoretical model of frequency-dependent transmission (Mesoudi & Lycett, 2009) shows that, when popular traits were favoured, a few prevalent cultural norms would become dominant – depending on the initial frequency of the traits – and exclude the remaining minority traits. Alternatively, when unpopular traits were favoured and popular traits were selected against, traits of intermediate frequency were expected to spread. However, this model does not take account of the functional variability of cultural traits and hence does not address the underlying evolutionary basis of such preferences.

**Figure 3.**
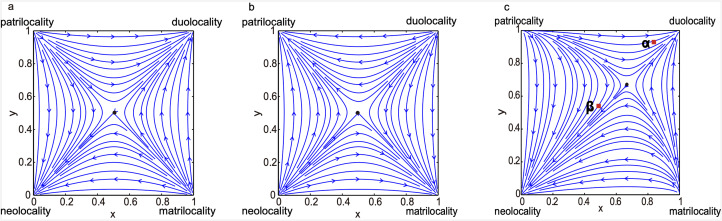
Modelling frequency-dependent evolution of post-marital residence, from Ji et al. (2016). The *x*-axis denotes the frequency of female dispersal and the *y*-axis the frequency of male dispersal in the local population. Arrowed blue lines denote directions of attraction. Black dots denote the unstable saddle points. (a) The boundaries (1,1) and (0,0) are locally asymmetrically stable, where the population within the basin of attraction of (0,0) will be attracted by the boundary neolocality, and the population within the basin of attraction of (1,1) will be attracted by the boundary duolocality. (b) The boundaries (1,0) and (0,1) are locally asymmetrically stable, where the population within the basin of attraction of (0, 1) will be attracted by the boundary patrilocality and the population within the basin of attraction of (1,0) will be attracted by the boundary matrilocality. (c) The boundaries (1,1) and (0,0) are locally asymmetrically stable and neolocal residence has larger basin of attraction. Red square *α* represents the proportions of Mosuo females and males who stay in their natal household after marriage in 16 Mosuo villages around matrilineal Pumi villages in Yongning in the 1950s. Red square *β* represents the proportions of Mosuo females and males staying after marriage in five villages in Lugu Lake Town in 2007.

One study incorporated empirical data to examine the decline of duolocal post-marital residence (i.e. when neither sex disperse) over time in part of southwest China (Ji et al., 2016). They showed that cultural norms of marital residence can evolve as a frequency-dependent strategy in response to changing fitness payoffs and starting conditions. Using asymmetric evolutionary games models to account for differing payoffs of post-marital residence types, they showed that the co-existence of two types is evolutionarily unstable, and one phenotype would eventually prevail. Minority strategies are unlikely to be maintained in the long term and the direction of cultural transitions depends on the initial frequency of strategies adopted by others in the population (Figure 3). A change in the local frequency could be enough to cause a norm to change even if payoffs remain unchanged. Immigrants may adopt the cultural norms of those around them, as happened when a village of patrilocal Pumi migrated into a Mosuo area in the nineteenth century, where duolocal residence was practised: over time they adopted the Mosuo kinship systems including duolocal residence patterns (Figure 3c, alpha case). Alternatively, if immigrants are numerous enough, they can drive the rarer local norms extinct in the face of extensive contact, as appears to be happening with the more recent influx of Han (an ethnic group where females usually disperse at marriage) into Mosuo areas (Figure 3c beta case); duolocality is currently in decline in favour of neolocality (Ji et al 2016). Micheletti et al. also show how the starting condition can influence the evolutionary trajectory of certain sex-biased behaviours related to warfare and other forms of altruism, based on sex-biased costs and the benefits of dispersal (Micheletti, Ruxton, & Gardner, 2018).

Cultural continuity is maintained by stable equilibria of cultural behaviours in the local ecology. In addition to natural selection in the physical environment, humans respond to self-imposed selection pressures (e.g. increased population density, domestication of animals, each others behaviour) with cumulative cultural knowledge. Such self-imposed selection involves ‘niche construction’, organism-induced change in the environment in which organisms experience new conditions. Niche construction can counteract selection pressures of natural selection as self-imposed selection becomes the main force initiating behavioural changes (Odling-Smee, Laland, & Feldman, 2013). Comparative studies showed that cultural traits related to external environmental conditions (e.g. technology) change more slowly compared with those traits related to social structures (e.g. material culture), possibly because natural selection operates directly upon the former with fitness consequences, while the latter carries neutral survival value and may have multiple equilibria with similar effectiveness (Currie & Mace, 2014). Ecological cultural traits with immediate fitness consequences are less likely to change once the optimum equilibrium within the available resource base is reached (Mesoudi, Whiten, & Laland, 2004).

Demography has a considerable impact on the capability to evolve cultural practices collectively in response to environmental pressure (Powell, Shennan, & Thomas, 2009). In biological evolution, genetic drift is stronger in small populations, which allows the fixation of deleterious traits. Similarly, as statistical anomalies in small populations cause cultural drift, small and isolated cultural groups are more likely to suffer the stochastic loss of cultural traits (Henrich, 2015). Population size, social network structure and mobility of population determine how many cultural traits the population can sustain (i.e. cultural complexity). In biology, the Allee effect (Lande, 1993) describes the existence of a threshold size for the viability of biological populations. Similarly, rare languages are more likely to show evidence of decline than commoner ones. As languages become rare they become less attractive for people to learn and use, so rare languages will become even rarer and so go extinct. There is some evidence for a speaker size threshold of language survival (Amano et al., 2014). Historical and ethnographic records documented how geographic isolation and small population size reduce cultural diversity by drift. Ethnographers recorded how the canoe, pottery, bow and arrow, and circumcision disappeared from various islands of Oceania (Rivers, 2013). Some cases, such as the canoe, were attributed to the death of all members of the society who had the requisite skills to manufacture the artefact, but some, like circumcision, died out despite the continued survival of its former practitioners. In the case of Tasmania, geographic isolation from the larger Australian population caused by the rising sea level led to the drastic loss of technological complexity (e.g. bone tool, clothing, fishing) over millennia (Henrich, 2004). Similarly, cultural comparative studies showed that, among cultural groups on the Pacific islands, the complexity of marine foraging technology correlates with population size and rate of contact with other populations (Kline & Boyd, 2010). Although cultural complexity was also observed in small hunter–gatherer societies with a high degree of specialisation (Collard, Buchanan, & O'Brien, 2013), a larger population is likely to contain more skilled toolmakers who could improve the technology and prevent its degradation as conformity-biased learning is more likely to preserve the complexity in a larger population (Kempe & Mesoudi, 2014).

### Discussion

Population genetics-type models are useful to show how these individual decisions scale up over successive generations of cultural inheritance in large populations (Acerbi & Mesoudi, 2015). Studies modelling the process of cultural evolution as a series of directional cultural transmissions (Cavalli-Sforza & Feldman, 1981) constrained by social learning biases (Boyd & Richerson, 1985) rarely address (at least not explicitly) the heterogeneity within the group and fitness values of cultural traits. Cultural behaviours evolved in response to selection pressures in the local ecology and individual fitness values in the same cultural group can display large variation. Empirical behavioural data are crucial to understanding the motivations behind individual behaviours that collectively shape population-level cultural changes in real life.

Some of the promising avenues of studying cultural extinction at the micro-evolutionary level include: collecting longitudinal data on demography, social networks and frequencies of cultural practices in endangered cultures; investigating intergroup networks between the endangered culture and its neighbouring groups; and examining the long-term consequence of heightened extrinsic mortality in endangered cultures, how it potentially influences the life history strategies of group members and contributes to the disintegration of kinship and social networks. The collection of empirical data on the fitness landscape of cultural behaviours can also help inform theoretical modelling studies with more realistic model set-ups and parameter estimates.

## 4. Conclusion

At the macro-evolutionary level, genetic and archaeological records of ancient human populations indicate that differences in physical ecology constrained the initial expansion and migratory routes of first farmers. Cultural extinction in the Holocene mostly occurred following intergroup competitions related to agricultural expansions, warfare, conquest, population replacement/admixture and colonisation. Comparison of reconstructed genetic and linguistic lineages revealed a broad range of possible outcomes after two cultural groups came into contact. In most cases, the extinction of language and culture does not mean the extinction of the genetic lineage or physical death of all group members. High genetic heterogeneity of individuals attributed to the same cultural group shows that, even in prehistorical times, ideas could travel across group boundaries without the movement of people, although some cultural differences can survive such mixing, showing that diffusion alone is unlikely to be responsible for the extinction of ethnolinguistic groups.

Historical records and ethnographies concur with the pattern observed in phylogenetic histories that small foraging cultures did not always suffer extinction after contact with large pastoralist/farmer groups. Micro-evolutionary processes of cultural extinction are likely to vary greatly among different groups and should be assessed on a case-by-case basis. At a micro-evolutionary level, cultural extinction can embody decisions by individuals (voluntary or involuntary) to stop practising and stop passing on to the next generation the traditional ways of life that were once neutral or adaptive in the local ecology but had become unattractive or maladaptive or just impossible in a new environment. The fitness costs of maintaining traditional cultures can be captured by its tendency to increase mortality rates or reduce fertility rates. Decision-making at the individual level in response to the changing costs and benefits of cultural practices contributes to the dynamics of cultural evolution.

Cultural evolutionary change over millennia in our evolutionary history can be studied in a phylogenetic comparative framework that incorporates linguistic, ancient genomic, archaeological and palaeoclimatic data to inform our knowledge of the evolutionary history of ethnolinguistic groups. For change over the (relatively) short term (over decades or centuries), cultural extinction may be fruitfully studied in a behavioural ecology framework that examines fitness consequences of cultural behaviours, given constraints in the local ecology and social networks, to help understand why individuals would, willingly or unwillingly, shift away from traditional norms in response to new fitness payoffs in new socioecological conditions. Future research of cultural extinction at the macro-evolutionary level could seek to incorporate insights and empirical evidence from other disciplines to inform the comparative studies global cultural extinction process. Studies of micro-level cultural extinction could incorporate empirical data on the changing fitness landscape and the frequency-dependent costs and benefits in endangered cultures to understand how cultural traits and identities are lost or maintained.

## Data Availability

No data was collected or analysed for this review.
